# Sentinel optical and SAR data highlights multi-segment faulting during the 2018 Palu-Sulawesi earthquake (M_w_ 7.5)

**DOI:** 10.1038/s41598-020-66032-7

**Published:** 2020-06-04

**Authors:** Guillaume Bacques, Marcello de Michele, Michael Foumelis, Daniel Raucoules, Anne Lemoine, Pierre Briole

**Affiliations:** 1 Observatoire de Physique du Globe de Clermont (OPGC), University of Clermont Auvergne, Clermont-Ferrand, France; 2 Bureau de Recherches Géologiques et Minières (BRGM), Orléans, France; 3 Ecole Normale Supérieure (ENS), Paris, France

**Keywords:** Geophysics, Seismology, Tectonics

## Abstract

The main active tectonic structure in the western part of Central Sulawesi (Indonesia) is the left-lateral Palu-Koro strike-slip fault. Its offshore section was thought not to have broken during the M_w_ 7.5 Palu Earthquake on 28 September 2018, challenging the established knowledge of the tectonic setting at this location. Here, we use Sentinel-1 SAR interferometry to produce a map of the ground velocities in the area of the M_w_ 7.5 earthquake for the seven months following the 2018 earthquake. We show evidence of surface deformation along the western coast of the Palu bay, indicating that the Palu Koro offshore fault section might have contribute to or been affected by the earthquake. As the possibility of multi-segment ruptures is a high concern in the area because of the high seismic and tsunami hazard, we present here, a fault model that includes the offshore section of the Palu-Koro fault. Thanks to four independents space-based geodetics measurements of the co-seismic displacement (Sentinel-1 and Sentinel-2 correlograms) we constrain the 3D co-seismic ground displacements. The modeling of these displacements allows us to estimate the co-seismic fault slip amplitude and geometry at depth. At the end, we consider the multi-segment faulting scenario, including the offshore section of the Palu-Koro fault, as a plausible model to explain the submarine landslides and the tsunamis. This study also gives the opportunity to observe a super-shear earthquake in the context of a complex fault network and implies an increase in the probability of submarine landslides within the bay in the forthcoming years.

## Introduction

**Figure 1:**
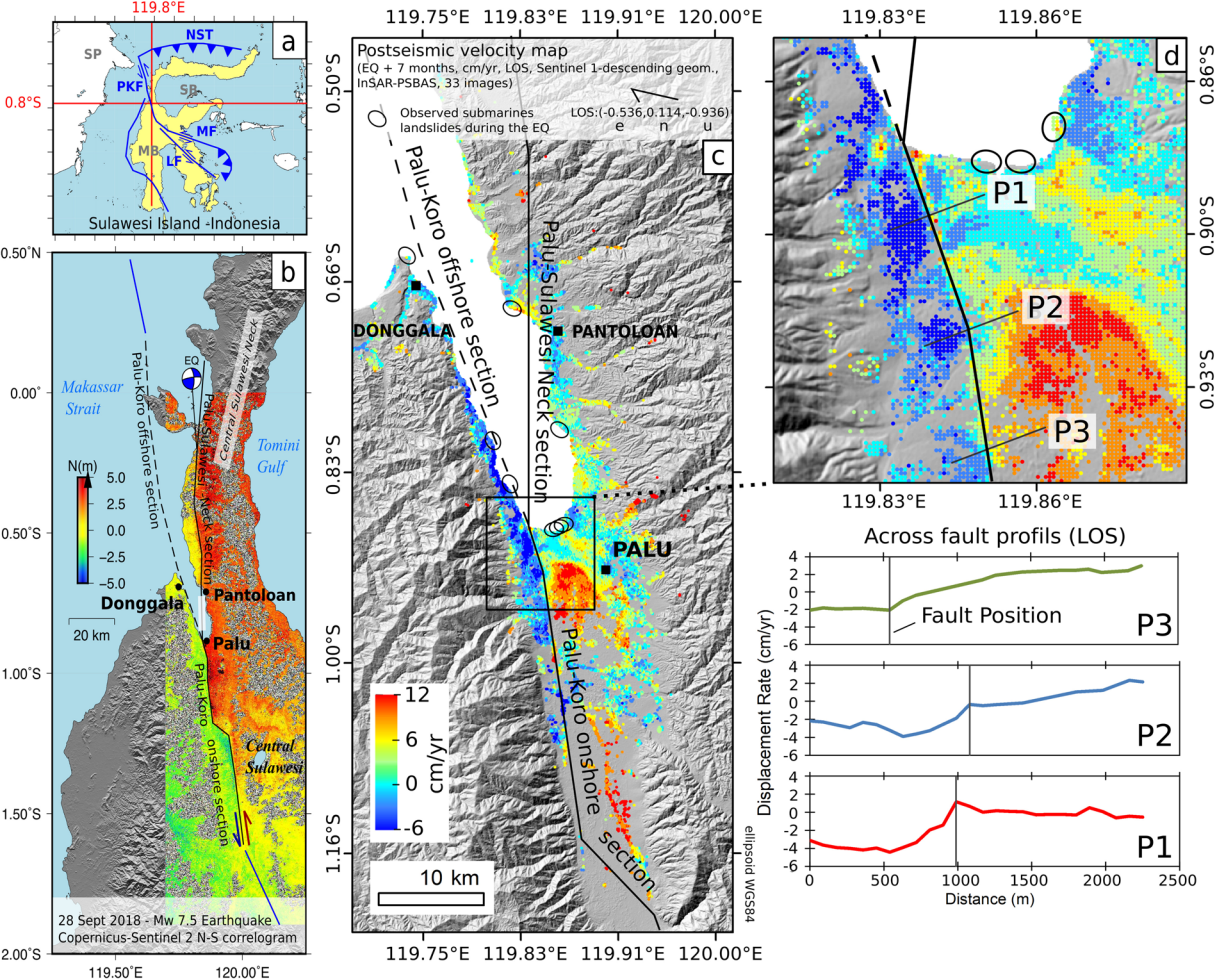
The 28 September 2018 Palu earthquake and its first seven months post-seismic velocity map. (**a**) simplified tectonic settings of the Sulawesi Island: NST, North Sulawesi Trench; MF, Mantano Fault; LF, Lawanopo Fault; PKF, Palu-Koro Fault; SP, Sunda Plate; MB, Makassar Block; SB, Sula Block. (**b**) North-component map of the co-seismic (northward: red, southward: blue). (**c**) Post-seismic line of sight (LOS) velocities during the first seven months from descending Sentinel-1 SAR interferometry. The line of sight unit vector is -0.526 east, 0.114 north and -0.936 up, and expressed from the satellite to the ground. (**d**) focus on Palu City. The surface projection of the faults model is presented by black continuous line for the PKF-onshore section & Palu-Sulawesi Neck (PSN) section and dashed black line for the PKF-offshore section (**b,c**). P1, P2, P3: across fault profiles of the post-seismic LOS velocity (cm/yr). The small ellipses in (**c,d**), indicate the location of the observed submarine landslides triggered by the earthquake as reported in ref. ^5,13^. Figure generated with Generic Mapping Tools (GMT 5.1.2, http://gmt.soest.hawaii.edu)^35^.

On 28 September 2018, a large tsunamigenic earthquake (M_w_ 7.5) struck Sulawesi Island (Indonesia) near the Minahasa Peninsula^1,2^ (Fig. 1a,b). The earthquake caused massive damages in Palu City, including dramatic onshore gravitational instabilities, soil liquefaction and a deadly tsunami^3–7^. The Sulawesi island, in south-east Asia, is known to be a highly seismogenic area since it lies within a complex fault system where the Australian, Pacific, Philippine and Sunda Plates converge in an area of about 500 km diameter. The main active structure onshore in the western part of Central Sulawesi is the left-lateral NNW-SSE trending Palu-Koro strike-slip fault (PKF) that forms the boundary between the North Sula and Makassar micro-blocks^8,9^ (Fig. 1a). During interseismic periods, it shows a transtensive behavior characterized by the presence of a pull-apart structure in the area of Palu city^8^. Studies based on Global Navigation Satellite System (GNSS) have shown that motion on the Northern sector of the Sulawesi region is marked by a 40 mm.yr^−1^ left-lateral strike slip along the PKF^8,10–12^. Therefore, the PKF is thought to partially accommodate the resulting interseismic stress load as six major earthquakes occurred along this fault within the past century (1907, 1909, 1927, 1937, 1968 and 2012 - M_w_ 6.6)^13^ including at least two tsunamigenic ones (1927, 1968)^14^.

However, the Palu earthquake challenged the initial conception about the tectonic setting at this location. Indeed, it initiated at 10–20 km depth, north of the Central Sulawesi Neck (lat_0_ = 0.178°S, lon_0_ = 119.840°E)^1–3,9^ on a previously unknown N-S trending fault section, located a few kilometers away east of the offshore section of the PKF and crossing the Central Sulawesi Neck on its western side (later referenced in the text as Palu-Sulawesi Neck fault or PSN - Fig. 1a,b). Earlier studies have shown that the September 28^th^, 2018, faulting propagated roughly from North to South, at super-shear velocity, ending ~30 km south of the city of Palu, thus, cumulating in a total faulting length of ~130 km with 4 to 7 m along-strike slip detected at the surface near Palu city^3,9,15^.

In such a tectonic context, tsunamis, like the one of 2018, can result from a rapid change of the bathymetry either due to the motion of the crust and/or to landslides. They are more prone to happen with thrust or dip slip faulting as those mechanisms normally produce larger vertical displacements in large surface area than strike slip events. However, in some circumstances, strike slip earthquake can eventually generate tsunamis^16,17^ as for the case of the 28 September 2018 Palu event. On the basis of fault slip modeling, derived from space geodesic data and/or seismic source inversion, numerical simulations have been used to evaluate the possible origins of the tsunami^9,18–20^. Although the early source inversion indicate dominant strike slip, these simulations predict also a non-negligible dip slip component^9,20^ likely to make this earthquake more tsunamigenic than initially thought. These fault slip models predict a vertical co-seismic motion of the sea bottom in the range of -2 to 1 m along the section of the fault crossing the Palu bay which could be enough to fit both the fast-arrival and the front waveform observed at the Pantoloan tide gauge station^20^. However, to explain better the tsunami inundation amplitude observed in the city of Palu, especially on the eastern shore of the Palu River, the contribution of submarine landslides as additional sources has been explored^13^. Indeed, several reports mentioned submarine landslides along the Palu Bay shoreline concomitant with the earthquake^3,4,13,21^ (Fig. 1c,d). Those landslides, due to their vicinity with Palu city, may explain both the observed short time arrival (~2 min after the main shock at Pantoloan tide gauge station), and the local wave shape and amplitude and, consequently, the tsunami run-up, notably at the Palu river estuary^13,21^.

The geodetic and seismic data covering this earthquake provide significant insights allowing to draw a comprehensive faulting scenario able to explain well the genesis of the tsunami. In these scenarios the possible contribution of the PKF-offshore to the earthquake does not have to be considered despite the major earthquakes recording at this location^13,14^ and the faulting surface signature along the onshore section of the PKF (Fig. 1b). However, the map of the post-seismic velocity inferred from Sentinel-1 synthetic aperture radar (SAR) interferometry during the seven months following the earthquake (Fig. 1c,d) show evidences of surface displacement along the western coast of the Palu bay. This supports the fact that the PKF offshore section has been activated during the earthquake or during the seven months after. The possible multi-segment ruptures in this area is of major concern due to the surrounding fault network complexity and its high tsunamigenic hazard. It is therefore important, to address the possible contribution of the offshore section of the PKF to the September 28^th^, 2018, M_w_ 7.5 Palu earthquake. In this study, the effects of an alternative fault distribution, in which the offshore section of the PKF is added, is evaluated by comparing the simulation results with the simplified version of the fault distribution used in previous studies. In order to model the related fault slip amplitude and geometry at depth, subpixel offsets tracking (here called correlograms) from Sentinel-2 optical and Sentinel-1 SAR images were calculated and used.

## Observations

### Post-seismic surface velocity map

A post-seismic surface velocity map (Fig. 1c,d) was produced using 33 descending Sentinel-1 scenes acquired between October 2018 and April 2019. The computation was performed *via* the Parallel Small Baseline tools provided by the Geohazards Exploitation Platform^22,23^ (P-SBAS). The surface motion derived from SAR interferometry are projected along the Sentinel-1 line of sight (LOS). Two remarkable features appear in this map. First a 5 km x 5 km sharply delimited area exhibits a displacement rate of up to 10 ± 2 cm.yr^−1^ is observed in the city of Palu. It could be related to soil compaction processes either due to urban activities or to gravitational soil deformations following the earthquake soil liquefactions. Second, the west coast of the bay presents a LOS velocity of -4 ± 1 cm.yr^−1^ that slowly decreases northward to ± 1 cm.yr^−1^ and follows the contour of the PKF onshore section southward along its first 10 km from the PKF and PSN junction point. Three in-land cross-fault profiles (P1, P2, P3 - Fig. 1d) show a ~4 ± 1 cm.yr^−1^ velocity step within 0.5 to 1 km along profile baseline with respect to the fault position. However, for the profile P3, the soil compaction on the eastern side of the fault may exaggerates this estimation locally. The relatively large displacement velocities along the west coast of the bay could be related to shallow crust relaxation processes following the earthquake. Due to the proximity of the coast with the southern part of the PKF offshore section, the displacement rate might indicate that the PKF offshore section has been active in the months following the earthquake or even during the earthquake itself.

### Sentinel-1, Sentinel-2 co-seismic correlograms

**Figure 2:**
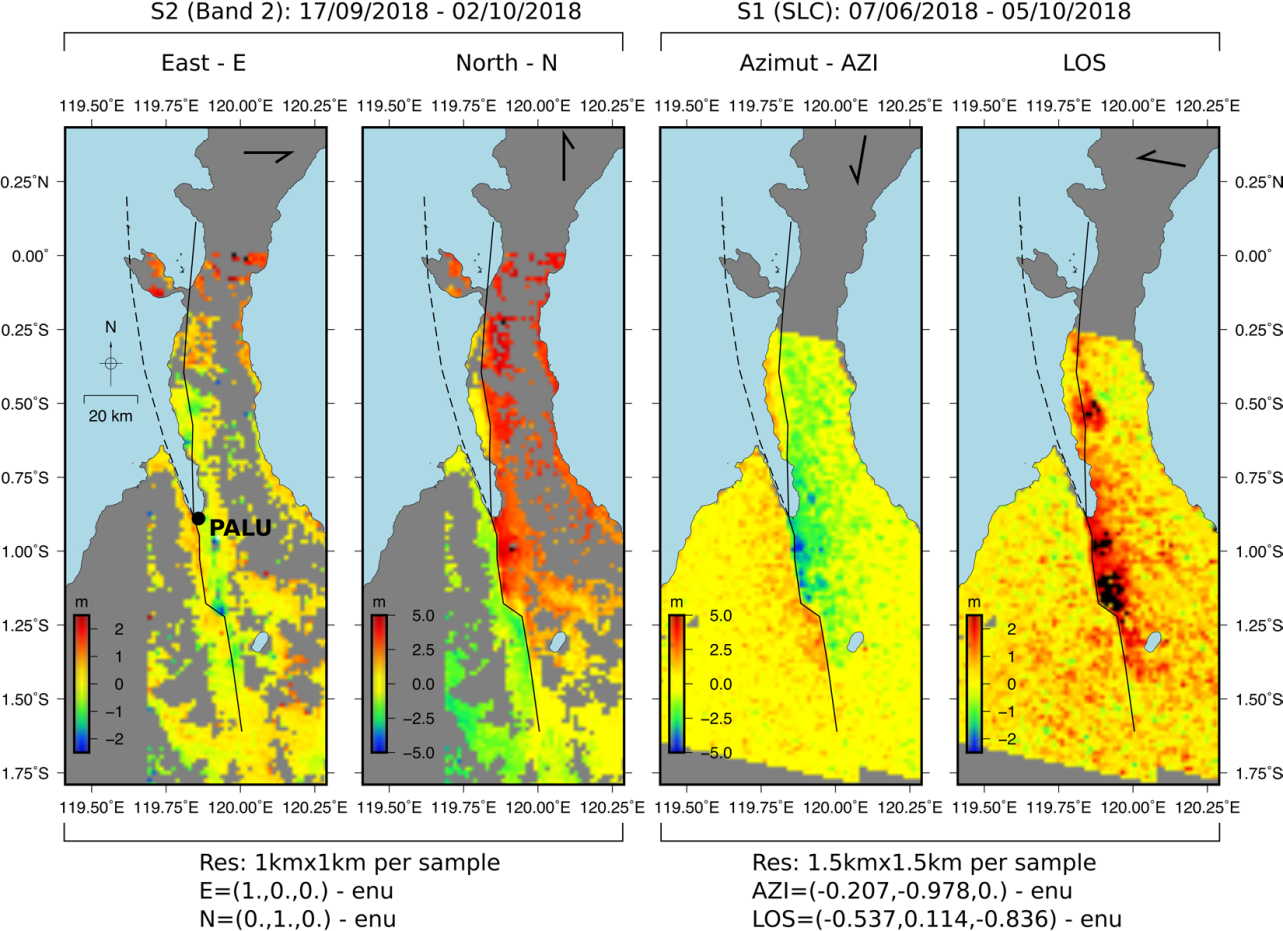
Down-sampled and filtered co-seismic correlograms. E, N, AZI, LOS stand for East, North, Azimuthal and Line Of Sight correlograms respectively. The top right arrow on each figure present the horizontal component of the displacement vector of each correlograms which east-north-up unit vectors (enu) are given in the bottom. The surface projections of the modeled faults are shown with black continuous line for the PKF-onshore section & PSN section and dashed black line for the PKF-offshore section. Maps were generated with Generic Mapping Tools (GMT 5.1.2, http://gmt.soest.hawaii.edu)^35^.

Four independent measurements of the co-seismic displacements were produced using image cross-correlation and offset-tracking techniques^24–28^. Optical images acquired by Sentinel-2 (band 2, 0.49 µm wavelength and 10 m resolution) were used to produce maps of displacement between the 17 September and the 02 October 2018 along the south-to-north direction as well as along the west-to-east direction (hereafter called N and E respectively - Fig. 2). The Single Look Complex (SLC) images acquired by Sentinel-1 on June 7 and October 5, 2018 provide, by correlation, two other displacement maps along the satellite fly path heading azimuth and in the LOS direction (hereafter called AZI and LOS - Fig. 2). The full-resolution N correlogram (Fig. 1a) shows dominant left-lateral dislocation and allows to precisely delineate the surface faulting path. The dislocation exceeds 6 m along the N direction with a sharp discontinuity running from ~60 km south of Palu city to the Palu bay. This is a proof that the fault rupture reached the Earth surface in this area. In comparison, northward, the displacement field is more diffuse for ~50 km along the PSN fault section, showing that the rupture may not reached the surface there with the same amplitude. We observe a total faulting length of ~140 km, which is consistent with previous studies^9^. The E correlogram does not show significant fault motion within the detection domain of the correlator procedure we used (~1 m i.e. 1/10^th^ of the Sentinel-2 pixel size). The AZI and LOS Sentinel-1 correlograms show similar trends as the Sentinel-2 correlograms although the observation geometries differ. The faulting path delineation is less sharp in these displacement maps. This may be due to the initial spatial resolution which, aside with the presence of speckle like noise, do not allow reaching the resolution of Sentinel-2. Nonetheless it is clear that, the displacement field reaches ~6 m along AZI and ~3 m along LOS, and the data spatial coverage is denser and more continuous than those of Sentinel-2 derived maps. While the Sentinel-2 correlograms (E and N) - mainly sensitive to horizontal displacements - indicate an almost pure strike slip motion, the LOS and AZI geometries, which are sensitive to both vertical and horizontal displacements, draw a more complex 3D surface deformation pattern. Thanks to their complementarity, the combination of Sentinel-1 and 2 correlograms is an asset to constrain, in a robust way, the 3D geometry of the co-seismic displacements as well as the landslides and soil compactions signals that may exist in some places.

### Models

**Figure 3:**
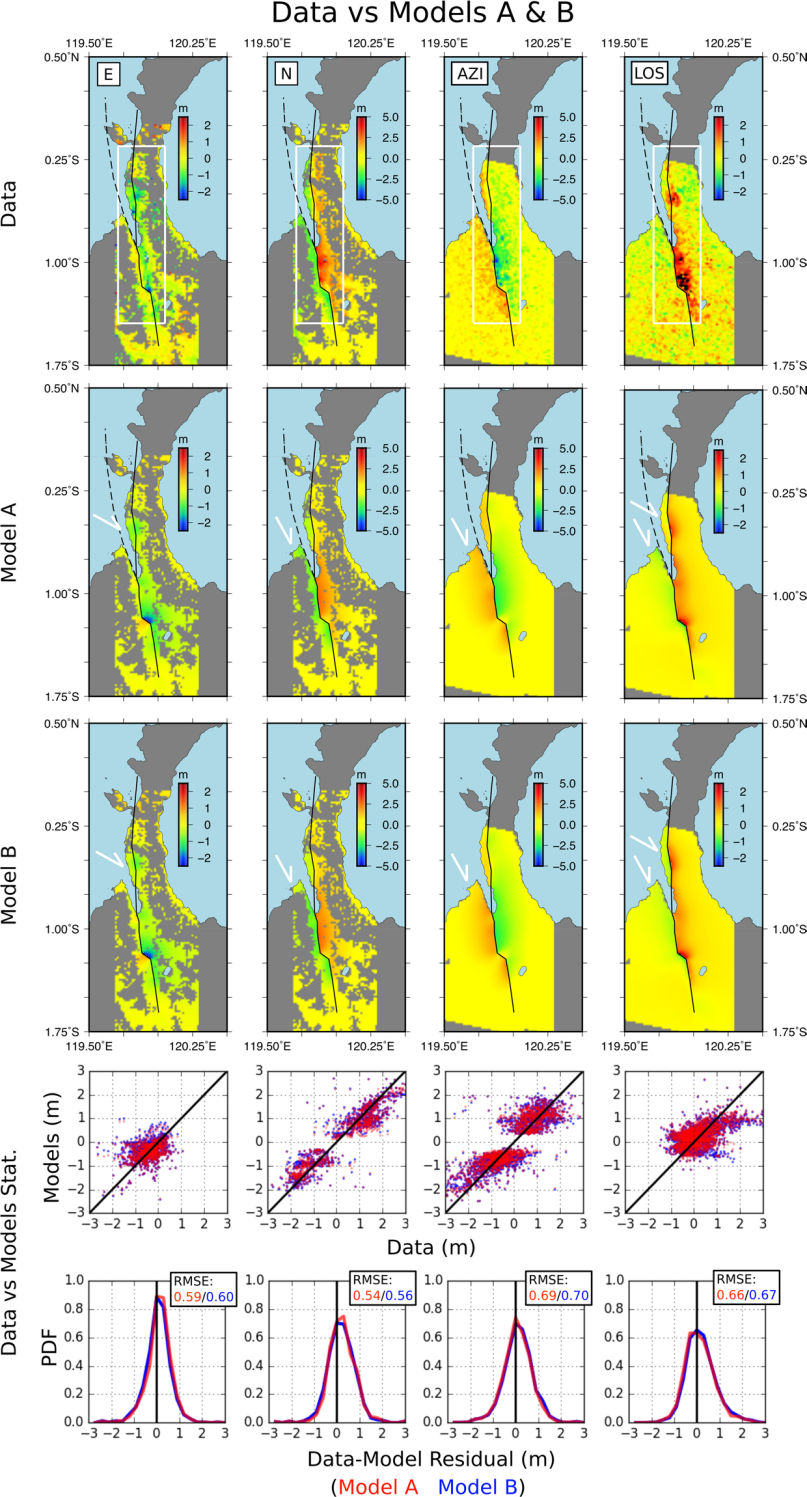
Ramp corrected data (first row), and the corresponding model predictions in E, N, AZI and LOS from the two faults model (A - second row) and single fault model (B - third row). The surface projection of the modeled faults are presented by black continuous line for the PKF-onshore section & PSN section, and dashed black line for the PK-offshore section. The two plots at the bottom show the data versus model statistics, model A being red coded and model B blue coded. Those statistics were evaluated for the region delimited by the white rectangle. RMSE stands for Root Mean Squared Error. Maps were generated with Generic Mapping Tools (GMT 5.1.2, http://gmt.soest.hawaii.edu)^35^.

**Figure 4:**
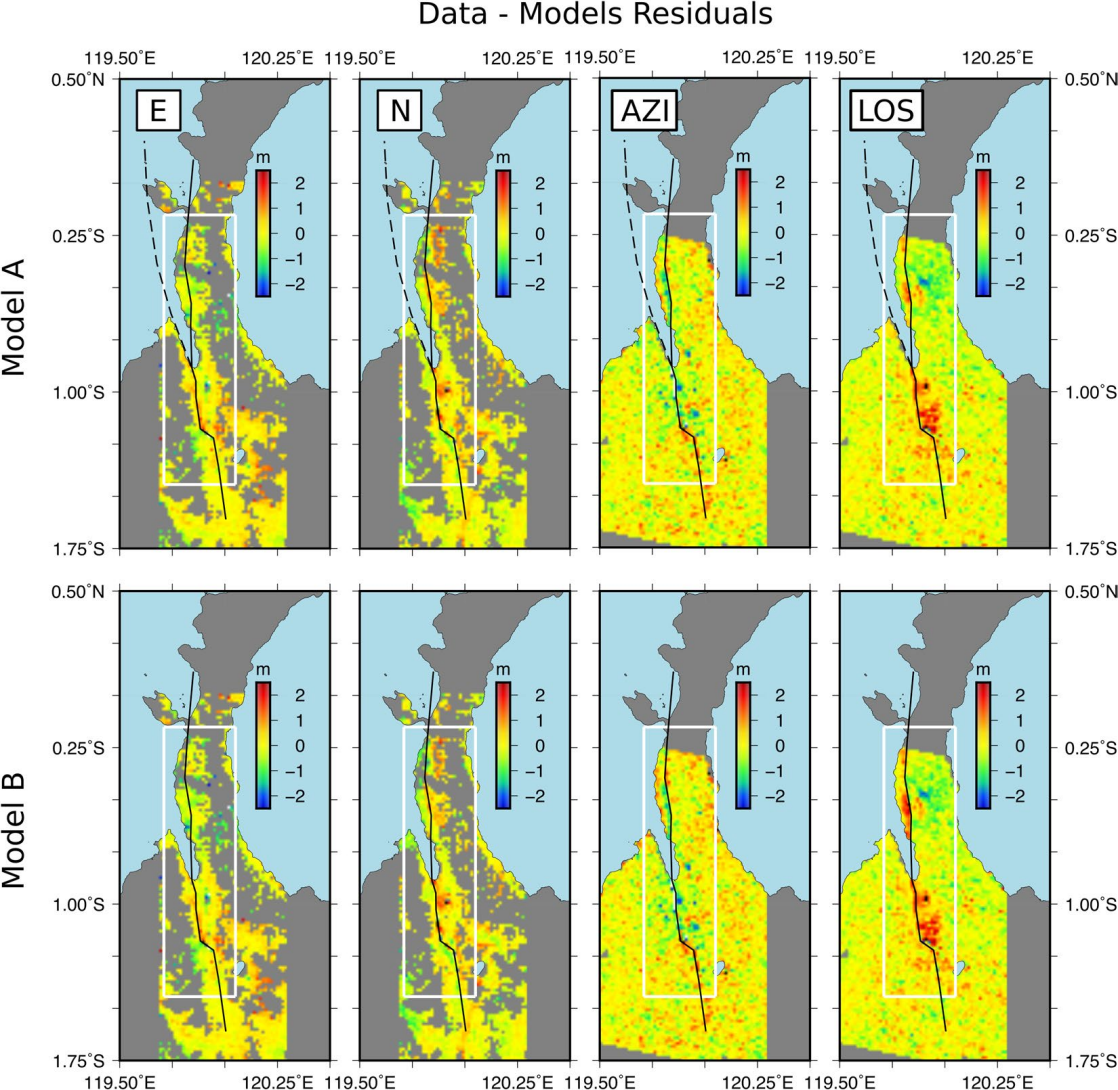
Data-model residuals in E, N, AZI and LOS for the two faults model (A - first row) and single fault model (B - second row) – Same nomenclature as Fig. 3. Maps were generated with Generic Mapping Tools (GMT 5.1.2, http://gmt.soest.hawaii.edu)^35^.

We consider a two faults distribution with simplified geometries to model the displacement maps (Fig. 3). In the one hand we consider the PKF onshore section and PSN section as a single continuous fault, in the other, the PKF offshore section (Fig. 4). The first model (model A) considers the two faults, the second (model B) only the PKF onshore and PSN sections. Contrary to the PSN and the onshore PKF sections which are clearly delineated from the N correlogram (Fig. 1b), the offshore section of the PKF remains poorly constrained as it lies undersea where there is no precise mapping of the fault. We used the tectonic settings map provided by Bellier *et al*.^29,30^ as a reference. It shows a fault surface path in the PKF area relatively similar to the one presented by Socquet *et al*.^9^. As a result, the offshore PKF section is a ~130 km long fault whose southern limit is connected to the onshore PKF in the city of Palu. It runs northward along the western coast of Palu bay with a −7.7° strike angle, and gets closer to the North direction toward the northern limit we arbitrarily set few kilometers westward the western coast of Sulawesi Neck (ending point: 0.20°N, 119.61°E) – Fig. 1b. For both models, we computed a fault slip distribution model a side with a 3D ramp correction model (longitude, latitude, displacement). From this point in the text, data are considered as 3D ramp corrected (Fig. 3). Details of the inversion procedure can be found in the methods section.

Both model A and B fit fairly well the observed displacement maps (Fig. 3). In both cases the final Root Mean Square Error (RMSE) between model and observation data appears very similar for the four types of deformation maps (E, N, AZI, LOS) and it ranges within reasonable values from 0.59 to 0.70 m (Fig. 3, statistic tables). The residual maps (Fig. 4) in LOS and N show discrepancies between observation and models mainly to the south of Palu city. This is likely due to the presence of landslides and soil compaction located here that produce large vertical and horizontal displacements not addressed by the Okada elastic dislocation model^31^ we employed in our modeling. The N correlograms data-model residual map also show a misfit around the northern part of the PSN section, as the positive displacement amplitude is underestimated by ~1 m in the models. As the N correlogram is mainly related to horizontal displacement, we guess that the difference is due to the fault geometry approximation at this location and/or a side effect of the model fusion procedure we used (see method section and SFigs. 1, 2). Differences between models A and B also exist north of Donggala city and on the eastern coast of the Sulawesi Neck with model A giving a fit significantly better at these locations (see white arrows in Fig. 3).

**Figure 5:**
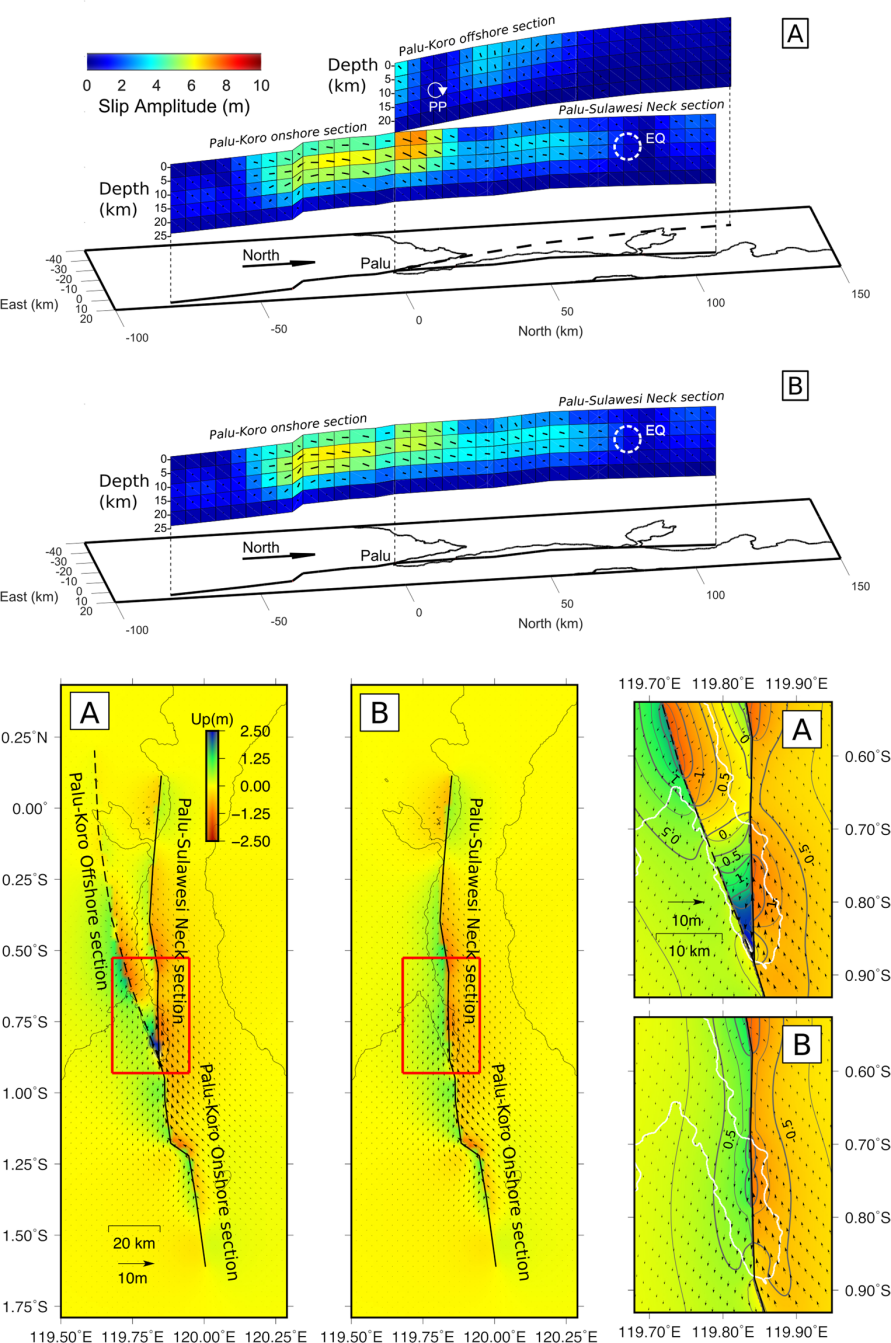
The 28 September 2018 Palu earthquake fault slip models in case of two faults model (A) and one fault model (B). Slip amplitude is color coded while the slip rake angle and amplitude are presented as arrows; when facing northward, the right foot block motion relatively to the left foot block. The white dashed circle represents the earthquake (EQ) hypocenter area. “PP” means Pivoting Point. Spatial coordinates present north and east distance from the city of Palu (km). Below, the surface displacement predictions from model A (left column) and model B (middle column). The vertical displacement is color coded while arrows present the predicted horizontal displacement. The surface projection of the faults model is presented by black continuous line for the PKF-onshore section & PSN section and dashed black line for the PKF-offshore section. The right column sub-pictures present details of the models predictions over the Palu bay (model A and B) - the coastline drawn in white. The corresponding area of interest are located by red rectangles in the left column (A) and middle column (B), dark-gray lines present vertical displacement iso-contours every 0.5 m for bold ones and 0.25 m for thin ones. Maps were generated with Generic Mapping Tools (GMT 5.1.2, http://gmt.soest.hawaii.edu)^35^.

When considering the two modelled fault slip distribution, differences that impact the predicted surface vertical deformations can be observed (Fig. 5). In both models, the slip distribution along the onshore-PKF & PSN fault extends northward up to the earthquake epicenter and ends southward 60 km south of Palu city, cumulating ~130 ± 10 km of faulting length and ~20 km in depth. The dominant faulting is left lateral strike, and oblique/dip slip faulting occurs in the shallower part of the PSN section. Looking at the fault slip amplitude distribution, we see the possible presence of slip deficit in the shallower part of the PSN section (0 to 10 km in depth) where we observe a dominant negative dip slip faulting mechanism. The strike slip amplitude might be under-estimated though, considering the misfit between model restitutions and the north displacement map at this location (second column Fig. 4). As the earthquake propagated southward, both models suggest that it initiated between 5 and 15 km in depth and reached the surface ~30 km north of Palu city. This is consistent with the previous interpretation of the displacement maps and early location of the epicenter. Model B is consistent with the model of Socquet *et al*.^9^ for the south to north and depth extension of slip patches as well as the amplitude and distribution of the strike slip component. However, there are significant differences when considering the dip slip component. They could be due partly to differences between modelled fault geometries, but we believe that they are also the consequence of the use of Sentinel-1 LOS and AZI data in our inversion that provide additional constraints to the 3D surface displacement. In the model A, the maximum slip is 6.5 ± 0.7 m near the junction point between the faults, and the offshore-PKF fault exhibits significant dip and oblique slip (up to 3 ± 0.5 m) behaving as a clockwise scissors fault - with a pivoting point located 15 km northward from the junction point and between 5 and 10 km in depth (Fig. 5). This faulting mechanism is surprising as the local inter-seismic stress loading geometry could be expected to favor a left–lateral strike slip faulting mechanism there. Model B yields vertical displacement predictions consistent with those of Ulrich *et al*.^20^, showing an uplift of the left foot side of the onshore-PKF & PSN fault, whereas the right foot side of the fault is subsiding (-1 m to 2 m within the bay). Model A predicts, however, a more complex vertical motions with a spatial distribution within the bay usually observed for trust or dip faulting earthquakes. In particular, it predicts a significant surface uplift ranging from 0.5 to 3 m south of the Palu Bay (Fig. 5).

As a comparison, the equivalent moment magnitude is M_o_ = 4.839 × 10^20^ N.m (M_w_ 7.71) for model A and M_o_ = 3.94 ×10^20^ N.m (M_w_ = 7.66) for model B (elastic shear modulus µ = 35 GPa), which is higher than previous estimations that predicted a M_w_ 7.5 earthquake^1,2^.

## Discussions-Conclusions

Our results suggest that by adding the offshore section of the Palu-Koro fault to the equation (i. e. model A) the model is statistically equivalent to the single-fault model (i. e. model B) but it features a more complex vertical/rotational displacement in the Palu Bay (Fig. 5). Our fault model A is simple but it is representative of the local tectonic settings as it encompasses both the major fault in this area (PKF) and the previously unknown PSN fault. Our modeling results (Figs. 4 and 5, Figs. 1–2) along with the post-seismic displacement map (Fig. 1c,d), suggest a faulting scenario implying that the 2018 Palu earthquake activated several fault strands, including the PKF offshore section. In this scenario, the offshore section of the PKF becomes a seismogenic source itself, capable of triggering a tsunami. This interpretation yields an alternative lead, tectonic oriented, to better understand the unexpected short time tsunami waves arrival and the presence of clustered submarine landslides along strike the offshore section of this fault.

Our model A suggests that block rotation and tilting occurred between two non-parallel fault strands (Fig. 5). According to it, motion on the PKF offshore section is predominantly along the dip, which might seem in contradiction with respect to the regional stress field predictions (mainly strike-slip geometry). The strike slip shear zone in the study area stems from the secular clockwise rotation of the Sula block^12^. This rotation generates transtension along the PKF^12,29,30^. Locally, GPS measurements campaign (between 1997–1999) reveal that there can exist some degree of evenly distributed convergence in a dominant strike slip regime. Therefore, strain is partitioned: up-dip motion might coexist with dip-slip motion if we consider that triggered motion on both PSN and PKF offshore sections generated a “tilt” of the block lying within the two faults. We also speculate that the previously unmapped PSN section might have partly accommodated the interseismic left lateral stress load initially thought to build-up along the Palu-Koro fault section only. From these interpretations and since it has been suggested that the earthquake ruptured at supershear velocity^9,15^, the 28 September 2018 Palu earthquake may be a rare example of a supershear earthquake involving multiple strands and faulting modes^32,33^.

Besides, another question raised by our study is the mid-term post-seismic behavior of the offshore section of the PKF. The way the fault system behaves post-seismically impacts the strain transfer along the overall fault system. For this reason, it is important to address the seismic/tsunami hazard assessment further by carrying a post-seismic deformation monitoring along the PKF and the PSN faults in the forthcoming years. Previous studies have shown the impact of submarine landslides triggered by the earthquake on the short time arrival and amplitude of the tsunamis observed along the Palu Bay. The Palu Bay is ~30 km long and 6–8 km wide and exhibits a maximum depth of ~700 m. Its bathymetry is shaped by the undersea flow of the Palu river and presents sharped and unstable quaternary sedimentary slopes, making this location very vulnerable to gravitational instabilities. Knowing if the post-seismic relaxation along the offshore PKF may contribute to ground instabilities over time and eventually trigger delayed submarine landslides along the Palu bay west coast is an issue that is worth of investigation.

A small fraction of the displacement may have occurred aseismically during the early stage of the post-seismic phase. Our data covers the co-seismic displacements, as well as the early post-seismic stage (~ 1 week after the main shock). This overlap between co-seismic and early post-seismic measurements does not affect our general understanding of the phenomenon and our conclusions. We simply highlight that a possible small fraction of early post-seismic displacement in our data might explain the greater seismic moment release estimated in this study compared with the one derived from seismology (M_w_ 7.5).

## Methods

### Cross correlation on Sentinel 1 and 2 data

The images acquired before and after the earthquake are primarily resampled to a common geometry using a Digital Elevation Model (DEM). Then, sub-pixel offsets are calculated within a moving window by estimating and interpolating the correlation peak. The residual offsets between both images is expected to result mainly from the surface deformation that occurred between the two acquisitions. The data we used (Fig. 2) result from sub-pixel image correlation on Copernicus Sentinel-2 (S2) and Sentinel-1 (S1) data (17/09/2018 - 02/10/2018 for sentinel 2 and 07/06/2018 - 05/10/2018 for Sentinel 1). For the S2 correlograms (band 2), the processing was performed with COSICORR^25^. We used a correlation window size of 32 pixels with a sampling step of 16 pixels. For S1 correlograms, we used the offset-tracking procedure implemented in the GAMMA processor^34^, more adapted for SAR image processing, with a 64 ×128 correlation window (64 in range - LOS direction, 128 along the AZI direction). The use of correlograms from both S1 and S2 is convenient since it provides four independent observations in relation with the 3D surface displacement associated with the earthquake. In the one hand, the horizontal displacement is provided by correlograms derived from S2 (E and N) and the correlogram derived from S1 in azimuth direction (AZI). In the other, the S1 LOS correlogram, contains contributions from both horizontal and vertical ground displacements.

### Data post-processing

The data are re-sampled into a regular 1 km × 1 km square grid for S2 and a 1.5 km × 1.5 km square grid for S1. This is done by computing a local mean around the node coordinates of the final grids, using the resampling tools from the generic mapping tools package^35^. Nodes with standard deviation (STDV) larger than 10 m are removed from the dataset as they are suspected to be not related to the tectonic signal, or poorly reliable due to high signal to noise ratio. At the end of the post-processing we get 10313 surface nodes for S1 correlograms (5158 nodes for the LOS grounds shift component and 5155 nodes for AZI grounds shift component). For S2, we get 13424 surface nodes (6679 nodes the E correlogram and 6745 nodes for N correlogram). Even if we get more surface nodes for S2 data compared to S1 data, they are more sparsely distributed (Fig. 2). The major loss of data is due clouds in the S2 images especially in the mountainous regions surrounding Palu city.

### Palu-Koro and Palu-Sulawesi Neck Faults

In our models we consider two faults: the Palu-Koro (PKF) onshore section as well as the Palu Sulawesi Neck (PSN) section as a single and continuous fault, and the PKF offshore section as a second fault. The PKF onshore section and the PSN fault have been designed on the base of the deformation map by following the surface fault trace (Fig. 1a). Since the earthquake is thought supershear^9,15^, which implies normally fault geometries with little complexities^32,33^, we chose to join the PSN and the PKF onshore section via a strait connection through the bay (Fig. 1b–d).

The PKF off-shore section has been delineated following these references^29,30^: it is a ~130 km long fault whose south limits is connected to the PKF onshore section at the Palu city location and runs northward along the Palu Bay west coast with a -7.7° strike angle. The strike direction gets progressively close to the north direction until the north limit defined at: (0.20°N, 119.61°E). We assumed the faults purely vertical with depth ranging from 0 to 20 km, and thus, varying only in strike direction.

### Data inversion

We divided the faults into smallest rectangular patches. The PKF onshore section and the PSN section (model B) cumulate 170 rectangular fault patches and the 2 faults system (model A) cumulates 285 rectangular fault patches. The fault patches size 5 km in depth and between 5 to 8 km in strike so every fault patch can be evenly connected with its proximal fault patch neighbors.

From the surface displacements derived from the correlograms we estimate the co-seismic slip distribution in two steps. First, we model the S1 and S2 data separately. Then, we fuse the two derived models into a single one that represents our final fault slip distribution associated with the earthquake. We repeat the inversion procedure for the two faults distributions (model A and model B) so we can compare the two surfaces displacements predictions. We used rectangular dislocation in elastic half space medium based on Okada’s analytics solutions^31^ to compute the reference functions. For each dataset (S1, S2) we estimate the strike and dip slip components of the fault slip, as well as a residual 3D ramp model, with LSQ based inversion method. A weighted smoothing operator (Laplacian) and constraints were also added into the inversion procedure. The bottom of the faults (at depth greater than 20 km) as well as the north limits and the south limits of the faults (with depth ranging from 0 to 25 km) are set to 0 m. In the case were the two faults are considered (model A), the south limit of the second fault branch is not constrained (that is, the first 20 km in depth). The optimal solution is chosen iteratively by varying the Laplacian weighting from 0.1 to 100 and choosing the solution that optimize both the RMSE (data-model) and the model roughness (known as L-curve criterion, eg^36^).

The model fusion is done by combining models (A or B) according to their respective model parameters variances. The model parameters variance is assessed by propagating the total estimation of the data - model RMSE. As the data-model residual distribution appears to be symmetrical, 0-centered with a gaussian like shape and as its spatial distribution seems to be random like (Fig. 3, Supp. Figs. 1 and 2), we chose to use it as a proxy for a posteriori estimation of the data initial covariance matrix. For a given fault patch we eventually get two independent estimation of the dip *or* strike co-seismic slip ‘m’ and the associated model parameters standard deviation ‘σ’: (m_1_, σ_1_) and (m_2_, σ_2_) for S1 and S2 data respectively. The final model (m_f_, σ_f_) result from the product of the two Gaussian shaped PDF, one per data set (S1, S2) and, consequently, defined as (Eqs. 1, 2)^37^:

1 $${m}_{f}=\frac{{\sigma }_{1}^{2}{m}_{2}+{\sigma }_{2}^{2}{m}_{1}}{{\sigma }_{1}^{2}+{\sigma }_{2}^{2}}$$

2 $${\sigma }_{f}^{2}=\frac{{\sigma }_{1}^{2}{\sigma }_{2}^{2}}{{\sigma }_{1}^{2}+{\sigma }_{2}^{2}}$$

As shown by Eq. (1) this model fusion procedure favors the model parameters (m) value with smallest variance, which corresponds to a better constrained estimation of the fault slip parameter. Model inversion and fusion dashboard can be seen in SF1 (model A) and SF2 (model B). As a final step, we proceed to the forward modeling in order to map, on a regular space grid, the 3D displacement with a dedicated attention to the offshore surface that represent the bathymetry changes due to the earthquake (Fig. 5).

## Supplementary information

Supplementary Figure S1.

Supplementary Figure S2.
