# Author Correction: Sentinel optical and SAR data highlights multi-segment faulting during the 2018 Palu-Sulawesi earthquake (M_w_ 7.5)

**DOI:** 10.1038/s41598-020-69652-1

**Published:** 2020-07-24

**Authors:** Guillaume Bacques, Marcello de Michele, Michael Foumelis, Daniel Raucoules, Anne Lemoine, Pierre Briole

**Affiliations:** 1 Observatoire de Physique du Globe de Clermont (OPGC), University of Clermont Auvergne, Clermont-Ferrand, France; 2 Bureau de Recherches Géologiques Et Minières (BRGM), Orléans, France; 3 Ecole Normale Supérieure (ENS), Paris, France

Correction to: *Scientific Reports* 10.1038/s41598-020-66032-7, published online 04 June 2020

In this Article, two References have been omitted for the Geohazards Exploitation Platform data processing tools. These are included below as References 1 and 2, and should be cited in the Observations section, under the subheading “Post-seismic surface velocity map” as below:

"The computation was performed via the Parallel Small Baseline tools provided by the Geohazards Exploitation Platform^22,23^ (P-SBAS)."

should read:

"The computation was performed via the Parallel-Small Baseline Subset (P-SBAS) service^1,2^ implemented on the Geohazards Exploitation Platform^22,23^."

Additionally, there is an error in the strike angle parameter of the Off-shore section of the Palu-Koro Fault. As a result of this, in the Observations section, under the subheading “Models”, in the first paragraph:

“It runs northward along the western coast of Palu bay with a − 7.7° strike angle, and gets closer to the North direction toward the northern limit we arbitrarily set few kilometers westward the western coast of Sulawesi Neck (ending point: 0.20°N, 119.61°E)—Fig. 1b.”

should read:

““It runs northward along the western coast of Palu bay with a − 20.9° strike angle, and gets closer to the North direction toward the northern limit we arbitrarily set few kilometers westward the western coast of Sulawesi Neck (ending point: 0.20°N, 119.61°E)—Fig. 1b.”

and in the Methods section, under the subheading “Palu-Koro and Palu-Sulawesi Neck Faults”, in the second paragraph:

“The PKF off-shore section has been delineated following these references^29,30^: it is a ~ 130 km long fault whose south limits is connected to the PKF onshore section at the Palu city location and runs northward along the Palu Bay west coast with a − 7.7° strike angle.”

should read:

“The PKF off-shore section has been delineated following these references^29,30^: it is a ~ 130 km long fault whose south limits is connected to the PKF onshore section at the Palu city location and runs northward along the Palu Bay west coast with a − 20.9° strike angle.”
