# Supplementary material for: Sentinel optical and SAR data highlights multi-segment faulting during the 2018 Palu-Sulawesi earthquake (Mw 7.5)
Source: Sci Rep. 2020 Jun 4;10:9103. doi: 10.1038/s41598-020-66032-7 (PMC7272429; doi:10.1038/s41598-020-66032-7)
Supplement: Supplementary file 2 — Supplementary Figure S2. [file 41598_2020_66032_MOESM2_ESM.docx]

**Sentinel optical and SAR data highlights multi-segment faulting during the 2018 Palu-Sulawesi earthquake (M_w_ 7.5)**

Guillaume Bacques^1*^, Marcello de Michele^2^, Michael Foumelis^2^, Daniel Raucoules^2^, Anne Lemoine^2^, Pierre Briole^3^

*Corresponding author: [guillaume.bacques@uca.fr](mailto:guillaume.bacques@uca.fr)
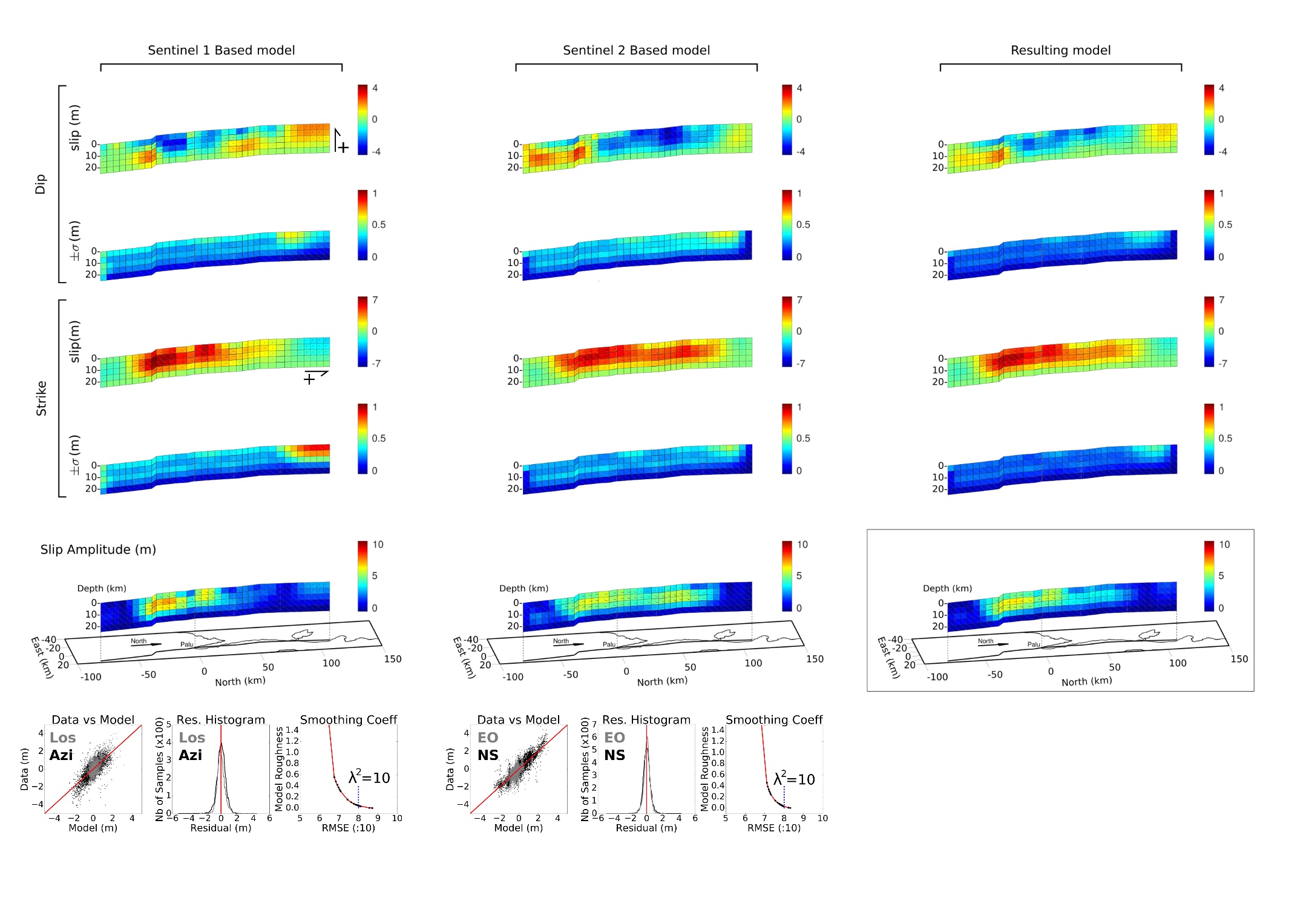


**Supplementary figure 2** **(SFIG2)**: Model computation dashboard in case when 1 fault is considered (Referred in the text as model B). First two lines present the Dip slip estimation and its associated model variance derived from the error propagation procedure. The third and fourth lines present the Strike slip estimation and the model variance. The fifth line present the slip amplitude. Data versus models statistical evaluation are presented in the last line. The first and second rows present the Dip and Strike slip estimation derived from the Sentinel 1 and Sentinel 2 correlograms respectively. The Third row present the resulting model we used for interpretation.
